# Transcriptome-Based Identification of Biomarkers Associated With Sphingosine-1-Phosphate Signaling Pathway in Aortic Dissection

**DOI:** 10.1155/ijhy/8882980

**Published:** 2025-10-15

**Authors:** Anmin Li, Xiu Chen, WenKao Huang, Ni Li, Linwen Zhu, Guofeng Shao

**Affiliations:** ^1^Department of Cardiovascular Surgery, Lihuili Hospital Affiliated to Ningbo University, Ningbo 315000, Zhejiang, China; ^2^Department of Thoracic Surgery, The First Affiliated Hospital of Anhui Medical University, Hefei 230000, China

**Keywords:** aortic dissection, CXCL5, ITGA5, sphingosine 1-phosphate

## Abstract

**Background:**

Aortic dissection (AD) is the most dangerous disease in acute aortic syndrome and is associated with serious complications. Current studies have shown that sphingosine-1-phosphate (S1P) has a certain effect on AD. Therefore, this study focuses on exploring biomarkers related to S1P in AD.

**Methods:**

Differentially expressed genes (DEGs) between AD and normal samples were identified from the GSE153434 dataset. Key module genes associated with the S1P score were then obtained using weighted gene coexpression network analysis (WGCNA). The DEGs were intersected with these key module genes to derive a set of intersection genes. Subsequently, a protein–protein interaction (PPI) network was constructed and screened to identify candidate genes. Further biomarker mining was performed through machine learning approaches followed by validation. Following this, gene set enrichment analysis (GSEA), immune infiltration analysis, investigation of regulatory mechanisms, and drug prediction were conducted. Finally, we quantified S1P concentration in human plasma using an ELISA kit, established an AD rat model, and validated gene expression levels using quantitative real-time polymerase chain reaction (qRT-PCR).

**Results:**

A total of 651 intersection genes were identified from the overlap between the 702 DEGs and 7108 key module genes. Subsequently, 20 candidate genes were screened, yielding two biomarkers: CXCL5 and ITGA5. Both biomarkers were enriched in the p53 signaling pathway, porphyrin and chlorophyll metabolism, and the NOD-like receptor signaling pathway. Furthermore, eight types of immune cells, including central memory CD4 T cells and natural killer T cells, were significantly elevated in the AD group compared with controls. ELISA quantification confirmed elevated S1P levels in human plasma. Additionally, utilizing an established AD rat model, we provided the first experimental validation that ITGA5 is highly expressed in dissected aortic tissue. Notably, CXCL5 exhibited the strongest significant positive correlation with central memory CD4 T cells. Regulatory network analysis revealed a relatively complex lncRNA–miRNA–mRNA interaction network. Finally, seven potential small-molecule drugs targeting ITGA5 were predicted, including cilmostim, cilengitide, and dimethyl sulfoxide.

**Conclusion:**

This study identifies ITGA5 as a novel biomarker for S1P-associated AD and reveals its potential underlying mechanisms and therapeutic candidates, providing a theoretical foundation for AD diagnosis and treatment.

## 1. Introduction

Aortic dissection (AD) is a severe cardiovascular condition where blood enters the middle layer of the aorta through a tear in its inner lining, creating a true lumen and a false lumen. This dissection extends longitudinally along the aortic wall. It is a critical emergency in cardiac and major vascular surgery, marked by a sudden onset, severe progression, and high mortality rate [1, 2].

The main clinical manifestation of the patient is a sudden onset of severe chest and back pain, often accompanied by hypertension. Depending on the degree of tearing, AD can involve multiple organs and systems. AD is classified into Stanford Type A or Type B based on the location of the tear. Stanford Type A involves the ascending aorta, requiring emergency surgical treatment regardless of whether the tear is located in the ascending aorta. Other types of dissection are classified as Stanford Type B, which can be managed with surgery once the dissection stabilizes.

If an AD ruptures and leads to massive bleeding, the patient will experience ischemic shock, and vital signs will rapidly disappear, eliminating the opportunity for surgical intervention. Currently, computed tomography angiography (CTA) of the thoracic aorta is the gold standard for diagnosing AD. Although this test is highly accurate, it can only diagnose the disease after it has occurred and cannot provide early prevention. Additionally, CTA carries risks such as kidney damage and allergic reactions to the contrast agent. Therefore, there is an urgent need to develop a method that can indicate an increased risk of AD before its onset, giving patients more time for emergency treatment and improving their survival rate [3].

To address this, we have conducted an in-depth investigation into the pathological mechanisms of AD. The pathophysiology of AD involves a complex interplay between mechanical stress, vascular degeneration, and inflammatory processes. Understanding these factors is crucial for early diagnosis and effective management. The aorta endures continuous blood flow pressure from the heart, with hypertension being one of the most common triggers. Hypertension increases the mechanical load on the aortic wall, making it more susceptible to dissection. The shear force induced by hypertension can cause a tear in the aortic intima, leading to the formation of a dissection. Degenerative changes in the aortic media can be caused by aging, genetic disorders, or other vascular diseases, such as Marfan syndrome, Loeys–Dietz syndrome, and Ehlers–Danlos syndrome type IV, which are connective tissue disorders. Additionally, inflammatory processes can also weaken the aortic wall. Chronic inflammation can lead to the degradation of extracellular matrix components, further compromising the structural integrity of the aorta. These mechanisms collectively reveal the complex pathological processes of AD, highlighting the importance of early intervention and personalized treatment [4–7].

The study by Pan et al. indicates that inhibiting the sphingosine-1-phosphate receptor (S1PR) 2 can prevent thoracic AD and rupture. This suggests that sphingosine-1-phosphate (S1P) may play a role in the development and progression of AD [8]. Lipids are renowned for their roles in energy storage and cell membrane formation. The structural diversity of membrane lipids ensures the flexibility and integrity of cell membranes necessary to adapt to various environments. S1P was discovered in the 1960s as the final product of sphingolipid metabolism [9]. It binds to extracellular partners, enriches in circulating fluids, and interacts with S1PRs coupled with G proteins to regulate embryonic development, postnatal organ function, and disease processes. Vertebrates possess five S1PRs (S1PR1–S1PR5) that respond to extracellular S1P, thereby regulating embryonic development, physiological homeostasis, and pathogenic processes in multiple organ systems [10].

Among the multiple S1PR isoforms, S1PR1 has been widely demonstrated to possess diverse protective effects on the vascular system [11]. For example, S1PR1 improves endothelial function, enhances endothelial barrier integrity, inhibits abnormal angiogenesis, and exhibits anti-atherosclerotic properties. S1PR regulates endothelial function by specifically activating S1PR1 in vascular isoforms, and its circulating levels serve as a potential biomarker of endothelial function [12]. In contrast, S1PR2 generally exhibits functions opposite to those of S1PR1. Studies have demonstrated that S1PR2 signaling plays a crucial role in various inflammatory diseases, such as atherosclerosis, acute vascular inflammation, cerebral ischemia/reperfusion injury, rheumatoid arthritis, and hepatitis. Furthermore, S1PR3 plays a positive role in angiogenesis, but its impact on endothelial barrier integrity and atherosclerosis is less fully understood. S1PR3 also plays a crucial role in protecting the heart from ischemia/reperfusion injury [8]. In contrast, S1PR4 is mainly expressed in the lymphatic system, while S1PR5 is more common in the immune and nervous systems. The expression levels of these two receptors are relatively low compared with S1PR13. In addition to S1PR2, the role of other S1PR subtypes in vascular function is also worthy of attention. For example, S1P-dependent calcium channels may regulate contractility through store-operated Ca2+ channels and S1PR2 in vascular smooth muscle cells, while the role in endothelial cells may be completely different. S1PR1 regulates changes in immune cell infiltration and extravascular coagulation by controlling vascular permeability. Activation of S1PR3 in endothelial cells can promote the production of nitric oxide (NO), thereby causing vasodilation, while in vascular smooth muscle cells, activation of S1PR3 causes vasoconstriction [13].

To this end, we conducted a series of bioinformatics analyses on sphingosine-1-phosphate-related genes (S1PRGs). We analyzed and validated differentially expressed genes (DEGs) involved in relevant pathways to assess their feasibility as potential biomarkers for AD.

## 2. Materials and Methods

All experiments conducted by us are carried out in accordance with the relevant guidelines and regulations. The data that support the findings of this study are available within the article and its Supporting Information (available here).

### 2.1. Data Acquisition

The AD-related datasets were mined from Gene Expression Omnibus (GEO) database [14]. GSE153434 (platform: GPL20795), containing ascending aorta tissue samples from 10 normal controls and 10 AD patients, was used as training set. GSE52093 (platform: GPL10558) included ascending aorta tissue samples from 5 normal controls and 7 AD patients and was applied as the validation set. Then, the 21 S1PRGs were downloaded from Pathway Commons [15] using the keyword S1P pathway.

### 2.2. Weighted Gene Coexpression Network Analysis (WGCNA)

To identify module genes associated with S1P, we employed WGCNA for a comprehensive analysis [16]. First, the single sample gene set enrichment analysis (ssGSEA) algorithm was used to calculate the S1P correlation score using GSVA [17], and the difference between the score in AD and control samples was compared by the rank-sum test. Then, all samples in the training set were subjected to cluster analysis and outliers were eliminated. Also, the optimal soft threshold was determined when R2 was 0.85 to construct the scale-free network. Subsequently, the adjacency matrix was converted into topological overlap matrix (TOM) and then hierarchical clustering was performed to determine the modules. The correlation between modules and S1P scores was assessed via Pearson, and the module with the highest positive correlation, *p* < 0.05, and |cor| > 0.4 was selected as the key module for subsequent analysis, and the genes contained therein were the key module genes.

### 2.3. Differential Analysis and Enrichment Analysis

The DEGs were obtained between AD and normal in training set with *p*.adj < 0.05 and |log2FC| > 2 using DESeq2 [18]. Then, the intersection genes were gained via overlap DEGs and key module genes. Afterward, enrichment analysis was applied to probe functions and pathways involved in intersection genes using clusterProfiler [19], including Gene Ontology (GO) and Kyoto Encyclopedia of Genes and Genomes (KEGG) (*p* < 0.05). Subsequently, in order to clarify the mechanism of intersection genes at the protein level, Search Tool for the Retrieval of Interaction Gene/Proteins [20] was used to construct the protein–protein interaction (PPI) network of intersection genes (confidence = 0.7). Furthermore, the gene network of the top1 module was selected by Molecular Complex Detection (MCODE) as the core network, and the genes contained in it were candidate genes. Default parameters were used: Degree Cutoff = 2, Node Score Cutoff = 0.2, K-Core = 2, and Max.Depth = 100.

### 2.4. Machine Learning

To obtain biomarkers of AD, we performed machine learning. Originally, least absolute shrinkage and selection operator (LASSO) analysis of candidate genes was performed using glmnet [21], according to lambda.min and ten-fold cross-validation, and then genes whose regression coefficients were not penalized to 0 were the characteristic genes. Then, candidate genes were screened by support vector machine recursive feature elimination (SVM-RFE) using caret [22] to obtain characteristic genes. Then, the results of the two parts were intersected to obtain candidate biomarkers of this study. Eventually, genes displaying similar expression trends with significant difference in the training and validation sets were identified as S1P-related biomarkers in AD.

### 2.5. Functional Enrichment of Biomarkers

To explore the pathways involved in biomarkers, we performed gene set enrichment analysis (GSEA). First, the samples of the training set were uncoupled high and low groups according to the expression of biomarkers. Then, limma [23] was used to analyze the difference between the two groups and calculate the log2FC. Afterward, GSEA was performed after sorting the log2FC from large to small (*p*.adj < 0.05 and |NES| ≥ 1). The top 5 pathways with the largest and smallest NES were selected for presentation. In addition, the genes coexpressed with the biomarkers were analyzed using GENEMANIA [24], as well as the functions they were involved in.

### 2.6. Immune Infiltration Analysis

To explore the effect of biomarkers on the immune microenvironment of AD, we first calculated the enrichment scores of 28 kinds of immune infiltrating cells in the training set samples using ssGSEA. Then, the Wilcoxon test was used to compare the differences between these cells in AD and normal groups. Subsequently, the correlation between biomarkers and differential immune cells was further analyzed.

### 2.7. Regulatory Mechanisms and Drug Prediction

In order to study the regulatory mechanism of biomarkers in AD, initially, miRNAs targeted by biomarkers were predicted using miRDB [25] and TargetScan [26] databases, and then the intersection results were obtained to obtain the intersection miRNAs. Then, lncRNAs corresponding to intersection miRNAs were obtained from starBASE database [27], and targeted lncRNAs were filtered according to clipExpNum > 4. Finally, lncRNA–miRNA–mRNA network was constructed. Furthermore, to mine potential AD drugs, the Drug–Gene Interaction database [28] was used to predict small molecule drugs corresponding to biomarkers. Eventually, these networks were visualized via Cytoscape [29].

### 2.8. Statistical Analysis

Bioinformatics analysis was performed in the R program. Data from different groups were compared using the Wilcoxon test. *p* < 0.05 was considered statistically significant.

### 2.9. Patient Enrollment and Sample Collection

This study was conducted at Li Huili Hospital of Ningbo Medical Center from December 2023 to April 2024. All participants provided written informed consent before enrollment. The study included 16 preoperative Type A AD patients as the experimental group and 16 healthy volunteers as the control group. Exclusion criteria for enrollment in the study were genetic syndromes associated with aortic diseases (such as Marfan syndrome, Ehlers–Danlos syndrome, Turner syndrome, Loeys–Dietz syndrome), a history of aortic trauma, pseudoaneurysm, prior aortic surgery, recent (< 1 year) cancer, and systemic diseases. Additionally, autoimmune diseases or blood disorders were excluded. Blood specimens were collected from patients in both the experimental and control groups. Samples were centrifuged at 3000 rpm for 15 min to obtain plasma, which was then used to measure S1P levels using an S1P ELISA kit (ELK8273, ELK biotech, Wuhan, China).

### 2.10. Animal Experiment Protocol

Thirty male SPF Sprague–Dawley rats, approximately 3 weeks old with similar weights, were selected and acclimated for 1 week. They were then randomly assigned into four groups using a completely randomized method: Group A (12 rats): received BAPN in drinking water + Angiotensin II (Ang-II) infusion at 1 μg kg^−1^ min^−1^ via osmotic minipump. Group B (6 rats): received BAPN in drinking water only. Group C (6 rats): received Ang-II infusion at 1 μg kg^−1^ min^−1^ via osmotic minipump. Group D (6 rats): blank control group. The method of administering BAPN in drinking water involved adding 1 mg/kg of BAPN. The drinking water was changed twice weekly. Before each change, the rats' weights were measured and their water intake was recorded. Each time the water was changed, the concentration of BAPN was gradually increased by 0.025% (starting from 0.1%, then 0.125%, 0.15%, etc.). The experiment lasted for 5 weeks. All rats had ad libitum access to food and water. Their food and water intake, behavior, and weight were regularly monitored. In case of rat mortality during the experiment, immediate autopsy was conducted to collect blood samples and preserve the entire aorta and heart. At the end of the experiment, surviving rats were euthanized using pentobarbital sodium injection to collect blood samples and isolate the entire aorta. Subsequently, the expression levels of target genes in blood and tissues will be assessed.

After verification in the previous experiment, 18 SPF male SD rats of 3 weeks old and similar body weight were selected and randomly divided into 3 groups: AD group (1 g·kg^−1^·d-1β-aminopropionitrile (3-Aminopropionitrile, BAPN) in drinking water + AngII 1 μg·kg^−1^·min^−1^ micro-osmotic pump group), intervention group (1 g·kg^−1^·d-1BAPN in drinking water + Ang-II 1 μg·kg^−1^·min^−1^ micro-osmotic pump group + cilengitide 853.32 mg·kg^−1^ intraperitoneal injection twice a week), and control group had normal diet and water. Aortic tissues were obtained for HE staining, Masson staining, protein blotting, immunohistochemistry, and immunofluorescence experiments.

## 3. Results

### 3.1. Identification of 7108 Key Module Genes Related to S1P Score

**Figure 1:**
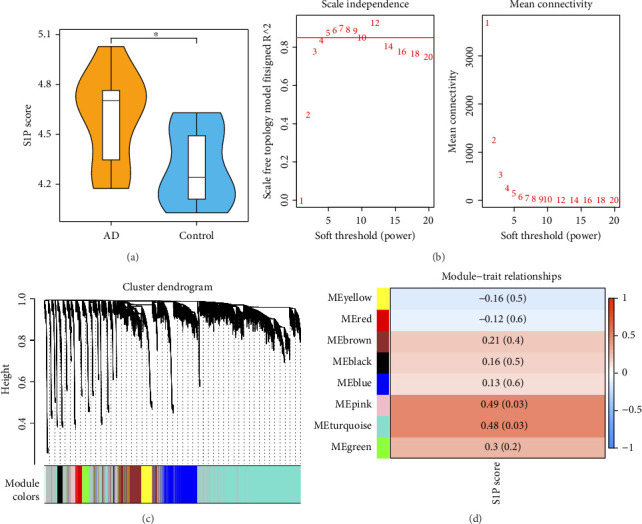
Differential analysis of S1P scores (a); soft threshold filtering (b); hierarchical clustering results (c); and key module screening (d).

There were significant differences in S1P scores between AD and normal groups (*p* < 0.05) (Figure 1(a)). Then, there were no outliers in the sample (Figure S1); the optimal soft threshold of 5 was chosen to construct the scale-free network (Figure 1(b)). Furthermore, in all, 8 modules were obtained (Figure 1(c)). Pearson showed that pink and turquoise modules had the highest correlation with S1P score and thus were used as key modules, which contained 7108 genes as key module genes (Figure 1(d)).

### 3.2. Functional Enrichment of 651 Intersection Genes and Identification of 20 Candidate Gene

**Figure 2:**
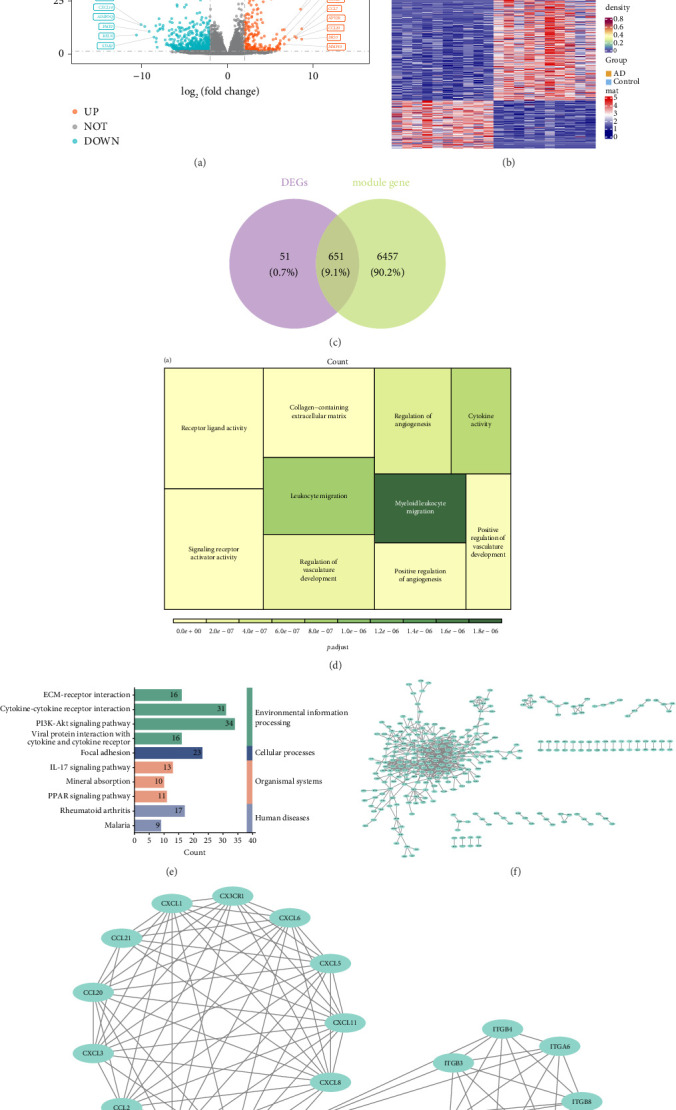
Heatmap (a); volcano plot (b); screening of differentially expressed S1P-related module genes (c); GO enrichment dendrogram (d); KEGG enrichment results classification diagram (e); PPI protein interaction network (f); and candidate gene network (g).

The 702 DEGs were gained between AD and normal, which contained 493 downregulated genes and 209 upregulated genes (Figures 2(a) and 2(b)). Then, the 651 intersection genes were obtained via overlap of DEGs and key module genes (Figure 2(c)). Furthermore, GO results showed that these genes were mainly enriched collagen-containing extracellular matrix, receptor ligand activity, and signaling receptor activator activity (Figure 2(d)), and the KEGG pathway indicated that these intersection genes were involved in the PI3K-Akt signaling pathway, focal adhesion, IL-17 signaling pathway, and rheumatoid arthritis (Figure 2(e)). In addition, the PPI network demonstrates complex interrelationships between intersecting genes at the protein level, such as CXCL8-CXCL5 and CXCL6-CXCL11 (Figure 2(f)). Afterward, 20 candidate genes were screened via MCODE for subsequent analysis (Figure 2(g)).

### 3.3. CXCL5 and ITGA5 Were Used as Biomarkers

**Figure 3:**
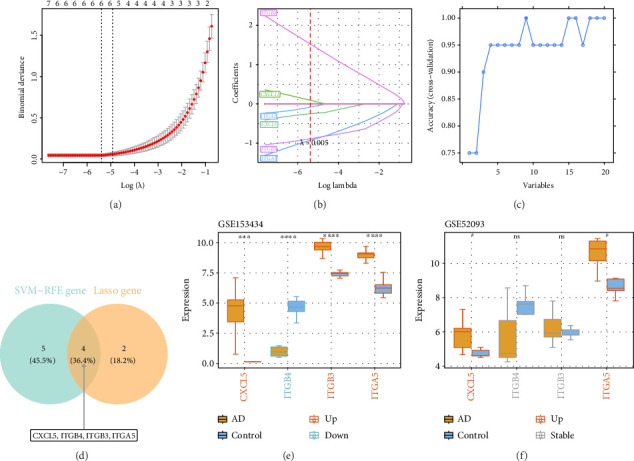
Ten-fold cross validation and coefficient spectrum of adjustment parameters in LASSO analysis (a-b); prediction of the true value change curve (c); screening of candidate biomarkers (d); expression of candidate biomarkers in the training set (e); and expression of candidate biomarkers in the validation set (f).

Among the 20 candidate genes, 6 and 9 characteristic genes were obtained by LASSO (Figure 3(a)) and SVM-RFE (Figure 3(b)) analysis, respectively. Then, 4 candidate biomarkers were gained via overlap of two results (Figure 3(c)). Furthermore, in the training set, CXCL5, ITGB3, and ITGA5 were significantly upregulated in the AD group, while ITGB4 was significantly downregulated in the AD group (Figure 3(d)). However, the expression trend of CXCL5 and ITGA5 in the verification set was consistent and remarkable with that in the training set (Figure 3(e)). Therefore, CXCL5 and ITGA5 were identified as biomarkers for this study.

### 3.4. Complex Functions and Pathways of Biomarkers

**Figure 4:**
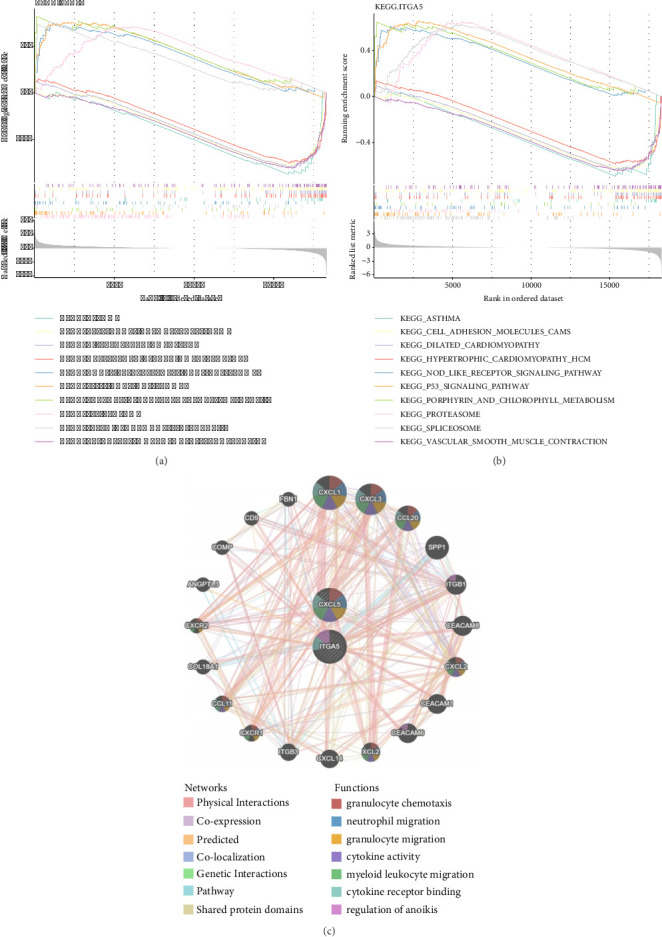
CXCL5 gene set enrichment analysis (a); ITGA5 gene set enrichment analysis (b); and interactions between genes (c).

GSEA suggested that the biomarkers were enriched with asthma, cell adhesion molecules (CAMs), dilated cardiomyopathy, hypertrophic cardiomyopathy (HCM), nod-like receptor signaling pathway, P53 signaling pathway, porphyrin and chlorophyll metabolism, spliceosome, and vascular smooth muscle contraction (Figures 4(a) and 4(b)). In addition, the biomarkers interacted with multiple genes, such as CXCL1 and CXCL3. There were many related functions among them, such as granulocyte chemotaxis, myeloid leukocyte migration, and regulation of anoikis (Figure 4(c)).

### 3.5. Correlation of Biomarkers With AD Immune Microenvironment

**Figure 5:**
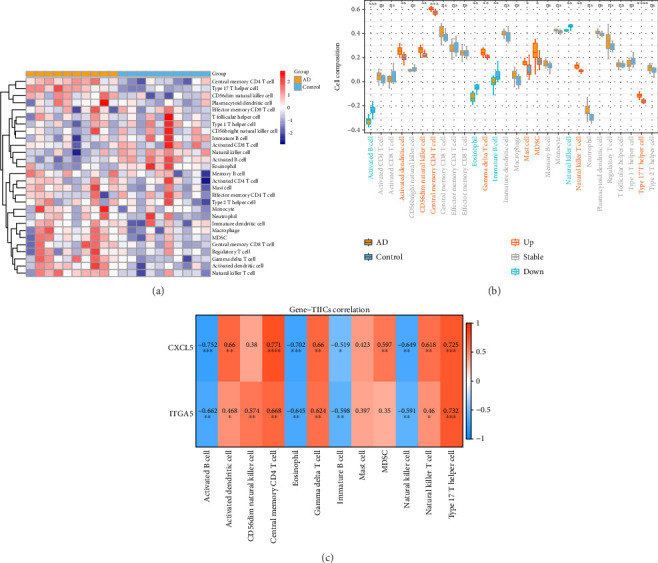
Immune infiltration heatmap (a); differences in the abundance of immune cells between diseased and healthy groups (b); and correlation between biomarkers and differential immune cells (c).

We evaluated the effects of biomarkers on the AD immune microenvironment.  Figure 5(a) shows the expression of 28 types of immunoinfiltrating cells in the sample. Activated dendritic cell, CD56dim natural killer cell, central memory CD4 T cell, gamma delta T cell, mast cell, MDSC, and type 17 T helper cell were markedly higher in the AD group, while the activated B cell, immature B cell, and natural killer cell were significantly lower in the AD group (Figure 5(b)). In addition, CXCL5 had the most remarkable positive correlation with central memory CD4 T cell (cor = 0.771), while CXCL5 had the most marked negative correlation with activated B cell (cor = −0.752) (Figure 5(c)).

### 3.6. Discussion of Regulatory Mechanisms and Potential Drug Targets in AD

**Figure 6:**
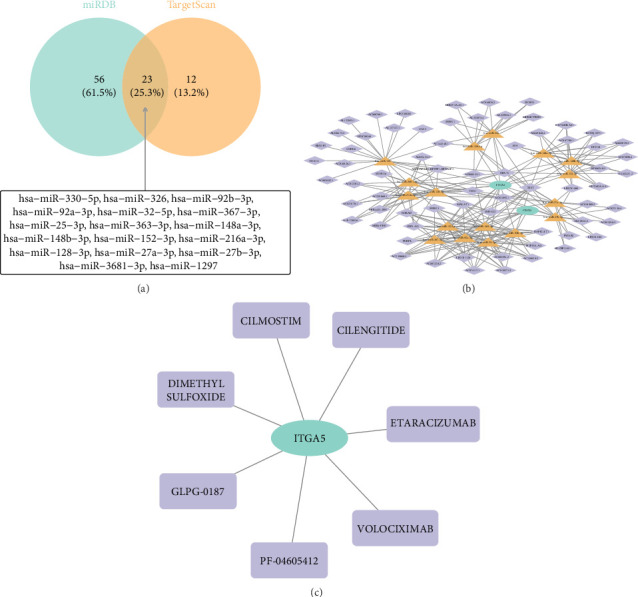
Screening of targeted miRNA (a); the network of 68cRNA–17miRNA–2 mRNA (b); and ITGA5 and its corresponding small molecule drug network diagram (c).

In order to explore the pathogenesis and regulation of AD, we constructed the lncRNA–miRNA–mRNA network. First, 23 intersection miRNAs were predicted jointly in miRDB and TargetScan database (Figure 6(a)), and then lncRNAs targeted by intersection miRNAs were predicted and screened in starBASE database. The network of 68lncRNA–17miRNA–2 mRNA was then constructed and visualized by Cytoscape (Figure 6(b)). ITGA5 was associated with hsa-miR-148a-3p and AC007780.1 to a certain extent, while CXCL5 was related to hsa-miR-363-3p and AC005394.2. Furthermore, we predict potential therapeutic agents for AD associated with biomarkers. ITGA5 predicted a total of 7 small molecule drugs in the DGidb database, while CXCL5 did not. The gene–drug network was constructed with 7 small molecules and ITGA5, including CILMOSTIM, CILENGITIDE, DIMETHYL SULFOXIDE, GLPG-0187, ETARACIZUMAB, PF-04605412, and VOLOCIXIMAB, suggesting that these small molecule drugs may be potential therapeutic targets for AD (Figure 6(c)).

### 3.7. S1P Plasma Levels

**Figure 7:**
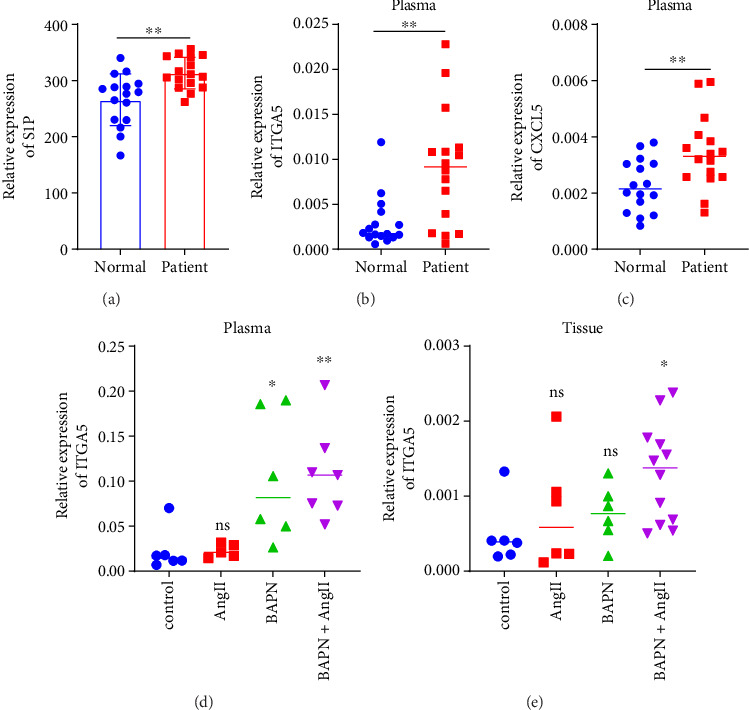
Relative expression of S1P in human plasma (a); relative expression of ITGA5 in human plasma (b); relative expression of CXCL5 in human plasma (c); relative expression of ITGA5 in rat plasma (d); and relative expression of ITGA5 in rat tissues (e).

The measurement results showed that in human plasma samples, compared with the control group, the plasma S1P levels of AD patients in the experimental group were significantly higher, and the difference was statistically significant (Figure 7(a)).

### 3.8. CXCL5 and ITGA5 Gene Expression Levels

In human and rat plasma samples, the expression of genes ITGA5 and CXCL5 was increased in the experimental group compared with the control group, and the same was true in tissue samples (Figures 7(b), 7(c), 7(d), and 7(e)).

### 3.9. HE and Masson Staining

**Figure 8:**
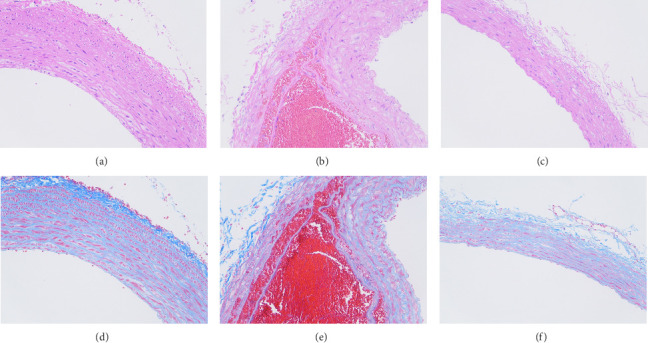
HE staining of the intervention group (a); HE staining of the AD group (b); HE staining of the control group (c); Masson staining of the intervention group (d); Masson staining of the AD group (e); and Masson staining of the control group (f).

HE staining showed that the nucleus was blue and the cytoplasm was red. Masson staining showed that collagen fibers were blue; muscle fibers, cellulose, and red blood cells were red. HE staining results showed that the distribution of cells between the aortic walls in the intervention group and the control group was denser, and the distribution of cells between the aortic tissue walls in the AD group was looser than that in the other two groups, and hematoma entering the aortic wall was visible (Figures 8(a), 8(b), and 8(c)). Masson staining results showed that the elastic fibers and muscle fibers in the aortic tissue of the AD group were disordered and broken, while the muscle fibers of the aortic tissues of the other two groups were intact (Figures 8(d), 8(e), and 8(f)).

### 3.10. ITGA5 Protein Expression Level

**Figure 9:**
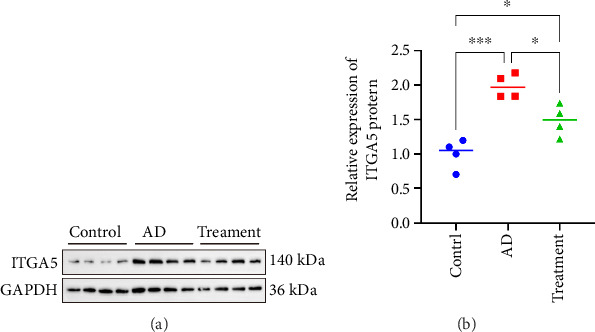
Electrophoresis bands (a) and increased expression of ITGA5 protein in aortic tissue of SD rat model (b). ^∗^Indicates a *p* value < 0.05. ^∗∗∗^Indicates a p value < 0.001.

Due to the limited number of lanes (16 lanes) of WB electrophoresis gel membrane, we randomly selected 4 samples from the control group, AD group, and intervention group to detect the expression level of ITGA5 protein. Western blotting experiments showed that the expression of ITGA5 protein was the highest in the AD group, followed by the intervention group, and the lowest in the control group. There were expression differences among the three groups (Figures 9(a) and 9(b)).

### 3.11. Immunohistochemistry and Immunofluorescence

**Figure 10:**
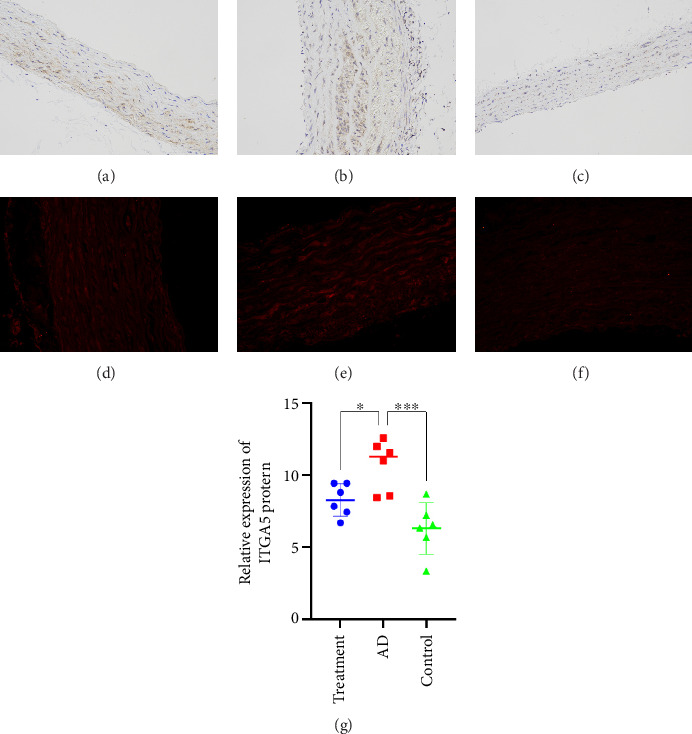
Immunohistochemistry of the intervention group (a); immunohistochemistry of the AD group (b); immunohistochemistry of the control group (c); immunofluorescence of the intervention group (d); Immunofluorescence of the AD group (e); immunofluorescence of the control group (f); and ITGA protein expression level (g). ^∗^Indicates a *p* value < 0.05. ^∗∗∗^Indicates a *p* value < 0.001.

Immunohistochemistry results showed that ITGA5 protein was mainly distributed in the cytoplasm or cell surface of aortic tissue but not in the nucleus. The level of ITGA5 protein in the AD group was higher than that in the intervention group and the control group (Figures 10(a), 10(b), and 10(c)), and the expression level was consistent with the WB experimental results (Figure 10(g)). Immunofluorescence also supports this conclusion, where red fluorescence indicates the expression position of ITGA5 protein (Figures 10(d), 10(e), and 10(f)). The difference in ITGA5 protein expression among the three groups can be seen from the red fluorescence intensity (AD group > intervention group > control group).

## 4. Discussion

AD is a critical and severe disease of the cardiovascular system. Early diagnosis or prevention can buy more rescue time for patients and greatly increase the survival rate. At present, there is no biomarker with good sensitivity and specificity for diagnosis in the early stage of AD. Therefore, we observed that inhibiting S1PR2 can reduce the incidence of dissection [8]. In order to explore the mechanism and find possible biomarkers, we conducted this study. Through bioinformatics analysis, we found two highly expressed genes in the S1P-related pathway, namely, CXCL5 and ITGA5, and tried to explore the possible mechanism of their role. Subsequently, their expression was verified in human and animal models through experiments. The possibility of ITGA5 as an early diagnosis of AD was preliminarily demonstrated.

### 4.1. Analysis of Biomarkers

The product of ITGA5 belongs to the integrin α chain family, and its encoded protein product is integrin α5. Integrins are heterodimeric integral membrane proteins composed of α subunits and β subunits and mainly play a role in cell surface adhesion and signal transduction in mammals [30]. ITGA5 mainly exerts its effects by regulating the downstream FAK/Src signaling pathway [31]. In the past, people mainly focused on the role of ITGA5 in tumor resistance, proliferation, migration, and other activities, and no research has focused on its mechanism and role in AD [32–34]. Previous studies have shown that ITGA5 is involved in the angiogenesis process in tumor tissues through the FAK signaling pathway [35]. This may indicate that in the pathophysiological process of AD, there is vascular repair and reconstruction, and ITGA5 plays an important role in this process, which needs to be confirmed by further research. If it can be proved that in the early stage of the pathological process of AD, there is vascular repair and generation under the ITGA5-mediated FAK pathway, then ITGA5 will serve as a potential biomarker for the diagnosis of early AD.

The protein product of CXCL5 belongs to the CXC subfamily of chemokines, also known as epithelial neutrophil-activating peptide, which is a chemokine mainly involved in the chemotaxis of inflammatory cells [36, 37]. CXCL5 promotes neutrophil migration and activates inflammatory responses through chemokine C-X-C motif receptor 2 (CXCR2) [38]. During AD, large numbers of neutrophils accumulate under the aortic intima until increased blood pressure causes intima rupture [39].

Noncoding RNAs have important biological functions such as regulating transcription and translation, and interacting with DNA, RNA, and proteins, and are closely related to the pathological process of cardiovascular disease [40]. We constructed a lncRNA–miRNA–mRNA network and found that miR-326 and miR-330-5p were closely associated with the expression of the ITGA5 gene. Studies have shown that inhibition of the ECM remodeling factor LOX downregulates ITGA5 and fibronectin, leading to the inactivation of downstream FAK/Src signaling. Overexpression of miR-326 and miR-330-5p directly targets ITGA5 and suppresses ITGA5 mRNA and protein levels, thereby inhibiting angiogenesis [41, 42].

### 4.2. GSEA

In this study, GSEA was performed on the two biomarkers and it was found that they jointly predicted many pathways, which were mainly enriched in the p53 pathway and vascular smooth muscle contraction. VSMC is the main cell in the aortic media. The proliferation and migration of VSMC play a vital role in the biomechanical properties of the aortic wall. It is reported that Talin-1 is mainly present in the aortic media and is significantly downregulated in AD aortic tissue. Further analysis confirmed that Talin-1 regulates VSMC proliferation and migration, ultimately causing pathological vascular remodeling, changing the structure and function of the vascular media, and leading to AD [43]. Luo et al. study demonstrated that BAPN + METH promoted apoptosis of aortic medial smooth muscle cells through the C/EBPβ-mediated IGFBP5/p53/PUMA signaling pathway [44]. Many genes, such as YAP1, Sirtuin-1, PCSK9, polycystin-1, and Brahma-related gene 1, are associated with the pathophysiological process of AD by affecting the proliferation and migration of VSMCs [45–47]. These research results are consistent with our analysis results.

### 4.3. Immune Cell Analysis

Expression of neuropilin-1 (Nrp1) on CD4 T cells promotes CD4 T cell trafficking into the aorta, and the stability and function of these cells appear to depend on the ligation of Sema3A with Nrp1 [48]. This suggests that CD4 T cells are involved in the formation of aortic wall inflammation. Literature suggests that preoperative lymphocytopenia, especially CD4+ T lymphocyte decrease through apoptosis, is associated with poor prognosis in AD patients undergoing surgery [49]. In the BAPN-induced AD mouse model, B cells infiltrated into the AD tissue, and plasma IgM and IgG levels were significantly increased. In addition, the expression of genes related to the B cell receptor signaling pathway was increased in the aortic tissue of BAPN-treated mice at the early stage of AD [50].

### 4.4. Analysis of Regulatory Mechanisms

We constructed a lncRNA–miRNA–mRNA network and found that miR-326 and miR-330-5p were deeply involved in the expression of the ITGA5 gene. Studies have shown that inhibiting ECM remodeling factors, LOX, downregulates ITGA5 and fibronectin and leads to the inactivation of downstream FAK/Src signaling and chemotherapy sensitization [31]. Overexpression of miR-326 directly targets ITGA5 and suppresses ITGA5 mRNA and protein levels [32]. miR-330-5p also inhibits ITGA5 [34].

### 4.5. Drug Analysis

We found 7 small molecule drugs that can act on ITGA5, which can be divided into two categories. One category is integrin inhibitors, including cilengitide, etaracizumab, GLPG-0187, PF-04605412, and volociximab, and the other category is drugs that act on the inflammatory process, including dimethyl sulfoxide and cilmostim. Considering that the product of ITGA5 is integrin α5, we plan to select integrin inhibitor drugs in the next experiment to verify the conclusions of this study and conduct further exploration.

## 5. Conclusions

In general, we found that ITGA5 may participate in the repair and generation of blood vessels in the pathological process of type A AD through the mediated FAK pathway. It is the first time that ITGA5 can be used as a biomarker for S1P-related AD, revealing the potential mechanism of AD and possible therapeutic drugs, providing a theoretical basis for the diagnosis and treatment of AD, and providing a direction for the next step of research. However, there are still some shortcomings in this study, including insufficient sample size and failure to verify the effects of related drugs, which are the directions for future improvements.

## Data Availability

The datasets generated and/or analyzed during the current study are available in the GEO database repository.

## Supporting Information

This article contains the following Supporting Information: An ethical approval document approved by the Experimental Animal Ethics Committee of Ningbo University; an ethical review document for biomedical research involving humans approved by the Medical Ethics Committee of Ningbo Medical Center Li Huili Hospital. In order to exclude the influence of outlier samples, we constructed a hierarchical clustering tree for all samples (*N* = 20). As shown in Figure S1, there are no outlier samples. All samples were used for subsequent analysis.
