# Supplementary material for: Transcriptome-Based Identification of Biomarkers Associated With Sphingosine-1-Phosphate Signaling Pathway in Aortic Dissection
Source: Int J Hypertens. 2025 Oct 15;2025:8882980. doi: 10.1155/ijhy/8882980 (PMC12543661; doi:10.1155/ijhy/8882980)
Supplement: Supporting Information — Additional supporting information can be found online in the Supporting Information section. [file 8882980.f1.zip › Animal Ethics Approval.pdf]

通知书

申请人 邵国丰

你提交的项目： 急性主动脉夹层动脉瘤外科诊疗技术创新与突破 （申请编号： 12732 ）中关于动物实验的方案，经我委员会审核，符合要求，予以初审通过。 动物实验时间为： 2023-11-20 ~ 2024-01-31

特此通知。

（注：此通知书仅限于实验方案使用，不得用作其他地方。）

宁波大学实验动物伦理委员会

2023 年 11 月 20 日
