# Supplementary figures and images for: Transcriptome-Based Identification of Biomarkers Associated With Sphingosine-1-Phosphate Signaling Pathway in Aortic Dissection
Source: Int J Hypertens. 2025 Oct 15;2025:8882980. doi: 10.1155/ijhy/8882980 (PMC12543661; doi:10.1155/ijhy/8882980)

# Sample Clustering and trait heatmap

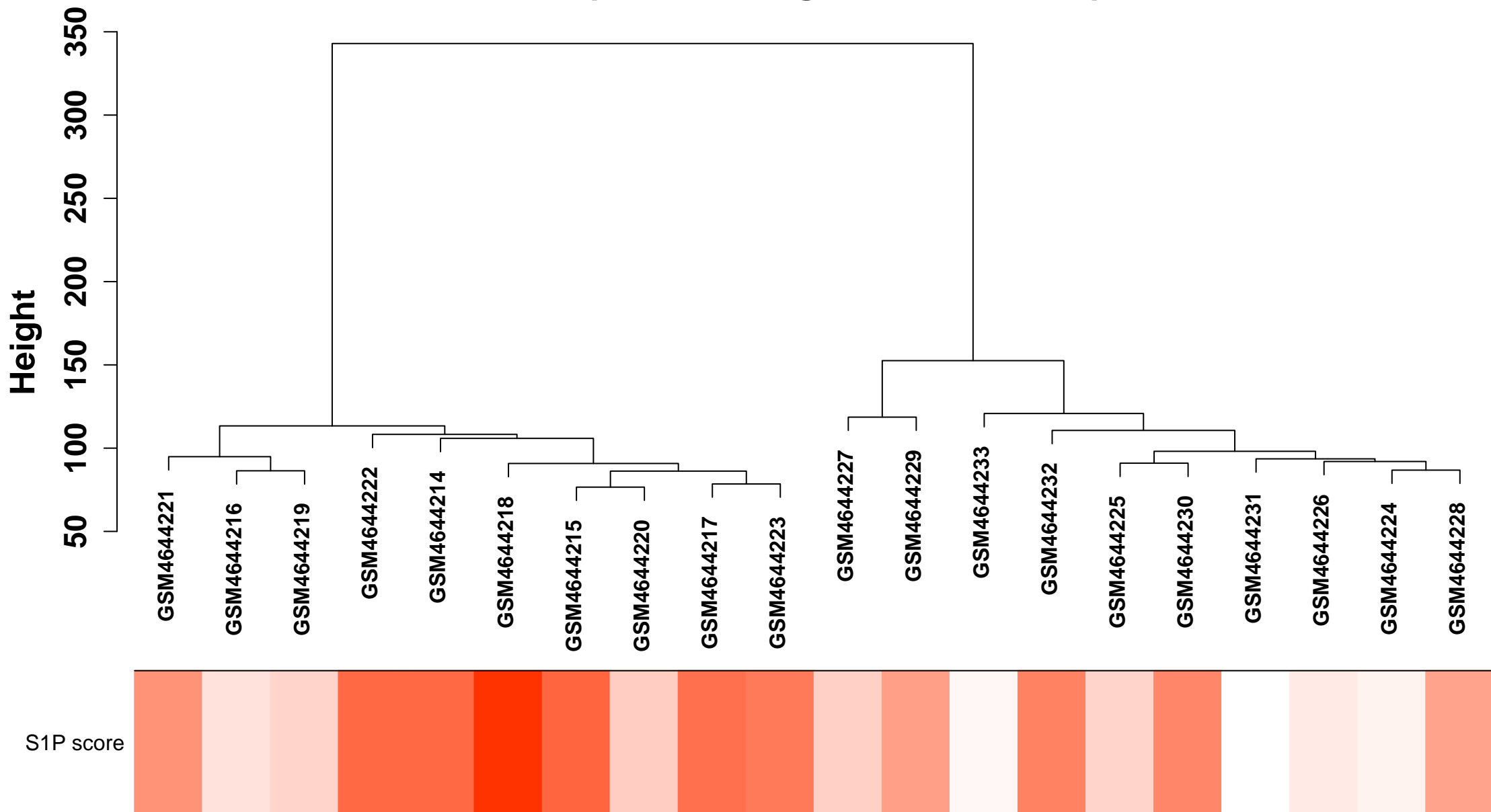

Supplement: Supporting Information — Additional supporting information can be found online in the Supporting Information section. [file 8882980.f1.zip › Fig.S1.pdf]
